# The advent of precision nutrigeroscience in cancer: from clinic towards molecular biology

**DOI:** 10.1016/j.jare.2025.08.034

**Published:** 2025-08-22

**Authors:** Zhijie Gao, Yunqing Liu, Yujie Cui, Yiling Han, Ke Cao, Qi Wu

**Affiliations:** a Department of Oncology, Third Xiangya Hospital of Central South University, Changsha 410013, China; b Department of Obstetrics and Gynecology, Hunan Provincial Maternal and Child Health Care Hospital, Changsha, Hunan, China; c Tongji University Cancer Center, Shanghai Tenth People’s Hospital, School of Medicine, Tongji University, Shanghai 200092, China

## Abstract

Nutrimental patterns have been deemed to have an impact on cancer development and the response to cancer therapy. Our growing understanding of cancer and host metabolism has highlighted that nutrient availability in the tumor ecosystem is a key factor in inhibiting tumor development. Subsequently, dietary interventions must take into account the specific characteristics of both the cancer and the host, which requires a detailed understanding of the mechanisms that determine the metabolic vulnerabilities in the tumor ecosystem. In this review, we provide an overview of various dietary regimens as interventions in both preclinical models and clinical studies. We discuss how dietary intervention can affect the homeostasis of the tumor ecosystem, including neoplastic, micro- and macro-environmental states that impact cancer progression and therapy. Our emphasis is on the prospects of precision nutrigeroscience, which involves developing individualized therapeutic approaches and predictors based on a thorough exploration of the mechanisms and critical factors. This approach has the potential to enhance the efficacy of anti-cancer treatments and prevention strategies.

## Highlights

• Precision Nutrigeroscience: A Novel Framework for Personalized Nutrition in Cancer.
• Dietary Interventions Modulate Tumor Ecosystem and Therapy Response.
• Microbiota-Targeted Diets Enhance Antitumor Immunity.
• Clinical Translation Requires Personalized and Biomarker-Driven Approaches.

## Graphical abstract

**Figure:**
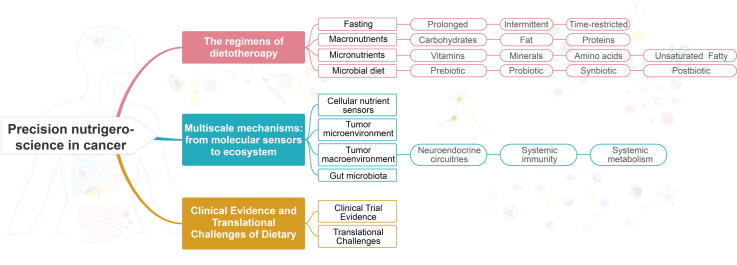

## Introduction

Cancer is a malignant disease that is influenced by a complex interplay of various factors. It is now advocated from an ecological perspective, where malignant growth is not solely dependent on abnormal cells but also on the interaction of multiple cell types within the tumor [1,2]. These cells are embedded in a framework of metabolic, neuroendocrinal, microbial, and immune circuitries. Additionally, aging, co-morbidities, and co-medications play a significant role in cancer development and progression [3]. Recently, there has been a growing belief that there has been a shift towards interventions that target the entire ecosystem rather than just eliminating cancerous cells. This approach has prompt the development of antiangiogenic therapies and immunotherapies, as well as the reversal of tumor-promoting processes such as dysbiosis and inflammation to impede tumor progression and enhance the outcomes of cancer treatment [4,5].

Epidemiological studies suggest that certain unhealthy dietary habits, such as consuming alcohol, excessive calories, highly processed foods, and large amounts of salt, sweeteners and *trans*-fatty acids, increase the risk of developing cancer [[6], [7], [8], [9]]. Therefore, the quality, quantity, and composition of diets determine the availability of nutrients in the bloodstream and affect the likelihood of developing malignant diseases. In recent, there has been growing interest in the metabolic diversity of cancer cells and infiltrating immune cells, and manipulating metabolic processes has become an eminently therapeutical approach [10]. In this context, dietary modification is widely recognized as a prevention tactic, as well as a complementary approach to conventional cancer therapy.

Nutrition influences tumorigenic processes by affecting body composition and organismal physiology through long-term modifications in macronutrient and micronutrient quantity and quality. Epidemiologically, various “healthy diets” such as the Mediterranean, vegan, fasting-mimicking, and ketogenic diets have been shown to have positive impacts on the manifestation of neoplastic disease [[11], [12], [13]]. As recent pre-clinical models developed, numerous reports have surfaced showcasing the impact of altering the availability of certain metabolites on the progress of cancer and its response to therapy [14]. Despite these promising findings, nutrition research still has methodological flaws, and there is a need for a future shift from empirical to precise approaches.

Here, we delve into the details of various dietary regimens and their effectiveness as either a prevention strategy or an adjuvant intervention in pre-clinical and clinical trials for cancer. Additionally, we describe the effects of nutritional interventions on the procarcinogenic ecosystem. Ultimately, we propose a novel concept called “precision nutrigeroscience”, which involves tailoring diet-based therapeutics to an individual's unique genotype, sex, age, and health-specific biomarkers. This personalized approach will lead to the identification of specific and effective regimens for cancer treatment.

In this context, we propose a novel conceptual framework: precision nutrigeroscience. This emerging interdisciplinary field integrates the principles of geroscience, cancer metabolism, and personalized nutrition to develop dietary interventions tailored to an individual’s tumor characteristics, biological age, metabolic status, microbiome composition, sex, and immune landscape. Distinct from traditional approaches in nutritional oncology or precision nutrition, which often address nutrient optimization or metabolic vulnerabilities in isolation, precision nutrigeroscience focuses on the interconnected axis of aging, metabolism, immunity, and cancer. Its goal is to delay tumor progression, enhance therapeutic efficacy, and counteract age-related immune decline. This framework is grounded in recent evidence demonstrating that both transient and chronic dietary restriction, selective modulation of macronutrients or micronutrients, and microbiota-targeted interventions can reshape tumor metabolism, modulate host immunity, and influence treatment responses. It recognizes that nutritional requirements and vulnerabilities are dynamic, evolving with aging and disease progression; that the gut microbiota and its metabolites (e.g., butyrate, inosine) critically influence tumor-immune interactions; and that immune rejuvenation and metabolic reprogramming can be strategically achieved through dietary means. By integrating multi-omics technologies and tumor–host profiling, precision nutrigeroscience aims to generate biomarker-guided, clinically actionable dietary regimens to be used as adjuvants or synergistic modalities alongside conventional cancer therapies.

## The regimens of dietotherapy in cancer

**Fig. 1:**
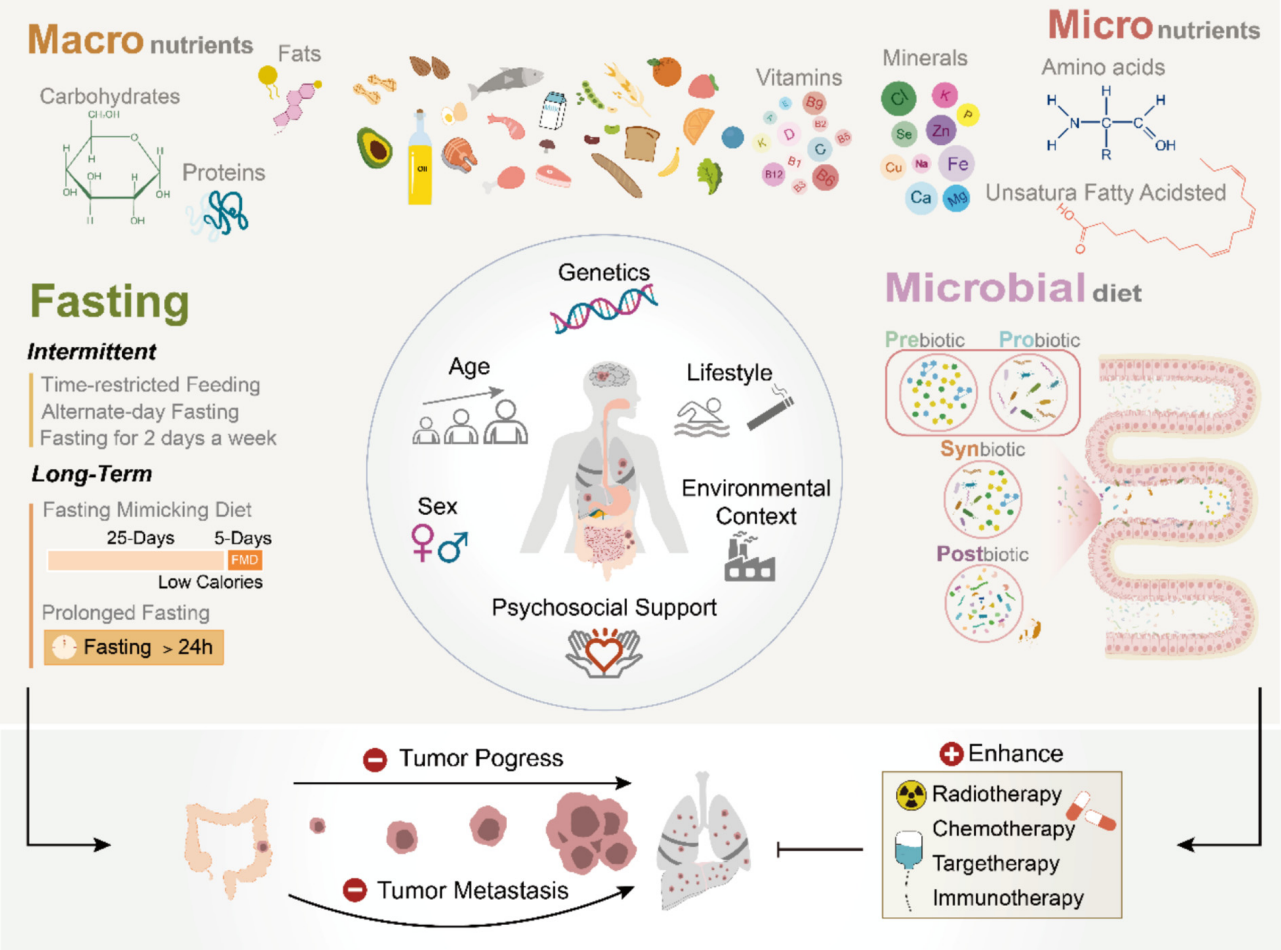
Dietary interventions and host factors collaboratively modulate tumor progression and therapeutic responses. This figure summarizes the integrative roles of dietary strategies and host factors in cancer modulation. Fasting protocols (intermittent, time-restricted, fasting-mimicking, and prolonged) suppress tumor growth and sensitize tumors to radiotherapy, chemotherapy, targeted therapy, and immunotherapy. Macronutrient modulation (carbohydrates, proteins, fats) and micronutrient supplementation (vitamins and minerals) affect tumor metabolism, immune regulation, and oxidative stress. Microbial-targeted diets (prebiotics, probiotics, synbiotics, postbiotics) remodel the gut microbiota to enhance immune responses and treatment efficacy. Importantly, host factors—such as genetics, age, sex, lifestyle, psychosocial support, and environmental context—interact with dietary interventions to shape anti-tumor outcomes. Together, these strategies provide a systems-level framework for dietary modulation in cancer therapy.

The consensus from both pre-clinical studies and clinical observations is that several dietary regimens have been found to have a beneficial impact on inhibiting cancer progression and enhancing the efficacy of cancer therapies (Table 1). Promising dietary strategies encompass fasting, interventions on macro- or micronutrients, and microbial diet (Fig. 1). Indeed, these nutritional interventions have the potential to optimize the physiological state to halt tumorigenesis.

**Table 1:** Classification of dietary interventions.

Class	Subclass	Description
Fasting	Classical	Fasting for random 2 days.
	Prolonged	Fasting for random over 2 days.
	Intermittent	Periods of fasting alternating with periods of eating.
	Time-restricted	Ad libitum feeding restricted to a specific time window per day.
	Caloric restriction	Reduction in daily caloric intake without malnutrition during the entire period of dietary intervention.
	Fasting-mimicking (FMD)	Cyclic caloric restriction where a low-calorie, ketogenic diet is provided during the restricted phase. FMD cycles are typically 3–4 days followed by 3 days of ad libitum diet in mice.
Interventions on macronutrients	Carbohydrate and glucose restriction	Carbohydrate intake is restricted typically under 25 % of the global caloric intake. Glucose restriction refers to specific restriction of glucose consumption rather than other forms of complex carbohydrates or sweeteners.
	Ketogenic diet	Strictly restriction of carbohydrates in order to induce ketosis, with increased fat and appropriate protein content. The fat ingredients including saturated fatty acid (SFA), monounsaturated fatty acid (MUFA) and polyunsaturated fatty acids (PUFA) vary in diverse studies in mice.
	Protein intervention	Reduction of dietary protein intake without alteration of the average caloric intake.
	Low-fat diet	Reduction of fat consumption to 20 % of calories and increase of vegetable, fruit, and grain intake.
Interventions on macronutrients	Vitamin/mineral intervention	Reduced or increased intake of vitamins and minerals.
	Amino acid intervention	Reduced or increased consumption of specific amino acids including methionine, tryptophan, serine, glycine, histidine, lysine, threonine and branched-chain amino acid (including valine, leucine and isoleucine).
	Fatty acid intervention	Specific restriction or supplement of fatty acids, like unsaturated fatty acid omega-3 PUFAs.
Microbiota-centered interventions	Prebiotics	Compounds which are indigestible by human enzymes and unabsorbed by the intestine, but benefit the activity and growth of gut microbiota.
	Probiotics	Orally-administered live microorganisms with the intention of colonizing the intestinal microbiota.
	Synbiotics	Oral formulation which contains complementary pre- and probiotics
	Postbiotics	Metabolites secreted by the microbiota exerting direct or indirect beneficial effects on the host.

### Fasting

Fasting or fasting-mimicking diet represent a nutritional strategy to reduce global caloric supply without malnutrition, including prolonged, intermittent or time-restricted fasting. In pre-clinical models, an alternative-day fasting (24-hour fasting periods alternating with 24-hour unrestricted feeding cycles) or intermittent fasting (including time-restricted feeding, 5:2 or twice per week fasting, and alternate day fasting) routine is becoming increasingly popular due to its numerous health benefits. This regimen involves fasting for one day and then eating without restriction on the following day. Recent studies have shown that this approach can delay the initiation of tumors, impede malignant growth promoted by obesity, and reduce the number of pulmonary metastatic foci. Additionally, alternate-day fasting, which involves fasting for 24 h and then having 24 h of unrestrained food intake, has been found to improve cardiometabolic parameters in human volunteers [15]. However, sustained alternate-day fasting exacerbates the cardiotoxicity caused by doxorubicin chemotherapeutics, suggesting that fasting period and patients with contraindications should be further taken into consideration [16]. Time-restricted fasting regimens (≥14 consecutive hours of daily fasting, with ad libitum feeding restricted to a 8–10 h window) have gained popularity as potential methods to protect against cancer in mice model [17]. However, fasting or caloric restriction requires careful calibration to ensure sufficient calorie restriction in absence of innutrition. Additionally, the Mediterranean diet is a recently developed eating pattern, with a predominant share of whole grains, fruit, vegetables, nuts, and fish-derived unsaturated fat. Recent study indicates that patients receiving a Mediterranean dietary pattern are sensitive to treatment with immune checkpoint blockade (ICB) and have an optimal progression-free survival [18].

### Interventions on macronutrients

Macronutrients can be divided into three main categories: carbohydrates, fat, and proteins. The distribution of these nutrients in diet has a significant impact on cancer progression.

Glucose is a crucial nutrient that tumours consume at high levels to fuel their growth. Dietary restrictions that lower glucose levels have been shown to be beneficial for cancer patients. The most commonly tested method is caloric or dietary restriction, but a better strategy may be an isocaloric diet that is low in glucose, such as the ketogenic diet. The ketogenic diet enriches in lipids but low in carbohydrates, and has been shown to reduce glucose levels in the blood. Ketogenic diets have been evaluated in clinical trials, which have shown that they can reduce body fat and tumor size, and prolong survival in patients with breast cancer in presence of a 3-month ketogenic diet combined with neoadjuvant chemotherapy [19,20]. Additional trials are being conducted to explore the potential benefits of a ketogenic diet in tumor development (Table 2). However, these studies have only included a limited number of participants, which has made it challenging to arrive at definitive conclusions regarding the effectiveness of the ketogenic diet. Furthermore, different types of fatty acids may have different effects, and the specific species of fatty acid can determine whether the ketogenic diet impairs tumor growth. The lack of a standard protocol for the ketogenic diet also makes it challenging to compare the results of different studies. While some studies have shown promising indications that ketogenic diet is viable and safe and enhance outcomes in cancer patients, further research is necessary. These studies should emphasize the importance of using a standard and clear protocol for the ketogenic diet.

**Table 2:** Clinical trials of dietary interventions in cancers.

Class	Subclass	Description	Cancer type	Treatment intervention	Phase	NCT number
Fasting	Caloric restriction	Over 15 % daily deficit of caloric intake with reduced fat and glycemic content + exercise	B-cell acute lymphoblasticleukemia	Chemotherapy	Phase 2	NCT05082519
		25 %-decreased caloric intake 1 week before radiotherapy and undergoing for 6–12 weeks	Breast cancer	Radiotherapy	Phase 2	NCT04959474
		50 %-decreased caloric intake 48–72 h prior to chemotherapy infusion + exercise	Breast cancer	Chemotherapy	Phase 2	NCT03795493
		Cyclic, 5-day, calorie-restricted (600 kcal on day 1; 300 kcal on days 2–5), low-carbohydrate, low protein diet every three weeks	Triple-negative breast cancer	Chemotherapy	Phase 2	NCT04248998
		Low-calorie diet 3 days before, during, and 2 days after chemotherapy	Breast/prostatecancer	Chemotherapy	Phase 2	NCT01802346
		Standardized 2-year caloric restriction	Breast Cancer	Preventive	Phase 3	NCT02750826
		Calorie-restricted plant-based diet for 6 dinners and 6 lunches per week + exercise	Breast Cancer	Chemotherapy	Phase 2	NCT04298086
	Short-term fasting	47–48 h short-term fasting prior to and 24 h after immunotherapy	Skin malignancy	Immunotherapy	Phase 1	NCT04387084
	FMD	4-day low caloric, low protein, vegetarian diet 72 h prior to and the day of neoadjuvant chemotherapy administration	HR+, HER2- breast cancer	Chemotherapy	Phase 3	NCT05503108
		8-week Mediterranean diet	Multiple cancers	Chemotherapy	Phase 1, Phase 2	NCT04534738
		5 days every monthly cycle for 6 cycles total in 6 months	Prostate adenocarcinoma	None	Phase 2	NCT05832086
		Plant-based, low-calorie (about 600 kcal on day 1; about 300 kcal on day 2 to 5), low-protein, low-carbohydrate diet	Breast cancer	Chemoimmunotherapy	Phase 2	NCT05763992
		5-day FMD regimen every 3 weeks, consisting of 700 kcal on day 1300 kcal on days 2–4, and 450 kcal on day 5	Advanced LKB1-inactive lung adenocarcinoma	Chemoimmunotherapy	Phase 2	NCT03709147
		Cyclic, 5-day, calorie-restricted (about 600 kcal on day 1; about 300 kcal on days 2 to 5), plant-based, low-protein, low-carbohydrate diet	Small cell lung carcinoma	Chemoimmunotherapy	Phase 2	NCT05703997
	Time-restrictedfasting	Fasting for over 16 h every night	Breast cancer	Chemotherapy	Phase 2	NCT05023967
		Time-restricted eating Monday through Friday only of each week	Prostate, cervical, or rectal cancers	Radiotherapy/ chemoradiotherapy	Phase 2	NCT05722288
Interventions on macronutrients	Low-carbohydrate diet	Low glycemic index food + moderate physical activity + high-dose vitamin D for 33 months after surgery	Breast cancer	None	Phase 3	NCT02786875
		Low carbohydrate diet/ ketogenic diet during treatment for 12 weeks	Breast cancer	Endocrine therapy /targeted therapy	Phase 2	NCT05090358
	Ketogenic diet	3:1 fat to carbohydrate + protein ratio for 5 days and 4:1 for 5 days	Glioblastoma	Chemotherapy	Phase 2	NCT04691960
		Continuous ketogenic diet from treatment onset (metformin 850–2550 mg/day)	Glioblastoma	Targeted therapy	Phase 2	NCT05183204
		4 −month ketogenic diet intervention	Glioblastoma	Chemoradiotherapy	Phase 1	NCT03451799
		Continuous ketogenic diet 7 days before and during treatment cycles	Follicular lymphoma,endometrial cancer	Targeted therapy	Phase 2	NCT04750941
		2-week ketogenic diet in combined with letrozole	ER + breast cancer	Endocrine therapy	Early Phase 1	NCT03962647
		18-week ketogenic diet intervention	Glioblastoma multiforme	None	Phase 2	NCT05708352
		Energy-restricted ketogenic diet to lower glucose and elevate ketones, combined with standard chemoradiotherapy	Glioblastoma	Radiatiotherapy/Chemotherapy	Phase 2	NCT01535911
		Modified Atkins diet (<20 g/day carbohydrate) to induce ketosis and enhance radiotherapy response	Glioblastoma	Radiotherapy	Feasibility	NCT03278249
		High-fat, low-carb diet plus KetoPhyt anti-inflammatory compounds; induces ketosis without calorie restriction	High-grade Glioma	None	Feasibility	NCT05373381
	Dietary Fat Intervention	Mediterranean diet supplemented with extra virgin olive oil vs. low-fat diet for relapse prevention	Early-stage Breast Cancer	Dietary education + EVOO / Low-fat diet	Non-phase	NCT04174391
	Protein intervention	Enriched high protein and high energy oral nutrition supplement	Colorectal cancer, non-small cell lung cancer	Chemotherapy/ chemoradiotherapy	Early Phase 1	NCT05648955
		Medical food restricting non-essential amino acids, combined with gemcitabine + nab-paclitaxel	Advanced or metastatic pancreatic adenocarcinoma	Chemotherapy	Phase 1/2	NCT05078775
		Medical food restricting non-essential amino acids, combined with FOLFIRI ± Bevacizumab	Metastatic Colorectal Cancer	Chemotherapy	Exploratory	NCT05183295
		Low-protein diet (10 % vs. 20 % protein) to modulate tumor immunity during immune checkpoint blockade	Solid tumors receiving immunotherapy	Immunotherapy	Feasibility	NCT05356182
Interventions on micronutrients	Vitamin/mineral intervention	Magnesium rich foods PO daily for 8 weeks	Lymphoma	None	Early Phase 1	NCT05294367
		16-week 300 mg capsules of the dietary supplement nicotinamide riboside + exercise	Acute lymphoblastic leukemia	None	Phase 2	NCT05194397
		12-week vitamin B6 PO daily	Prostate cancer	Antiandrogen therapy	Phase 2	NCT03580499
		6-month vitamin D3 (10000 IU/day) supplement	Basal cell carcinoma	Photodynamic therapy	Phase 1	NCT03467789
	Fatty acid intervention	Usual diet with the addition of two ounces of walnuts daily for 4–10 weeks	Prostate Cancer	Surgery	Phase 2	NCT03824652
		14-month 3 g/day of purified EPA capsules, taken once daily	Prostate Cancer	Surgery	Phase 2	NCT02333435
		Two capsules of 1000 mg fish oil daily (180 mg EPA and 120 mg DHA), or one capsule of vitamin D3 weekly (50000 IU)	Breast cancer	Chemotherapy	Early Phase 1	NCT05331807
		Letrozole 2.5 mg or/and Fish oil 2700 mg by mouth daily for 30 days	Breast cancer	Endocrine therapy	Early Phase 1	NCT02538484
		12-month 5 g/day of omega-3-rich fish oil including 4 g of purified monoglycerides EPA, capsules, taken once daily	Prostate cancer	None	Phase 2	NCT03753334
	Polyphenols	Low polyphenol diet for 4–6 weeks	Prostate cancer	Surgery	Phase 1	NCT01823562
Microbiota-centered interventions	Prebiotics	11-week isocaloric whole foods diet higher in fiber	Melanoma	Immunotherapy	Phase 2	NCT04645680
		15 capsules containing dietary fiber in psyllium husk per day	Multiple cancers	Radiotherapy	Phase 3	NCT04534075
	Probiotics	Clostridium butyricum CBM 588 probiotic strain PO BID during immunotherapy for over 12 weeks	Renal Cell Carcinoma	Immunotherapy	Phase 1	NCT03829111
	Synbiotics	Prebiotics and probiotics administration 1 week before, during, and 6–8 weeks after chemotherapy-radiotherapy	Anal squamous cell carcinoma	Chemotherapy/radiotherapy	Phase 2	NCT03870607
	BIG MACS diet	'BIG MACS Diet' and one protein shake per day for 4 weeks prior to and after surgery	Colon Cancer	Surgery	Phase 3	NCT05658263

Recent research suggests that high-sugar diets, particularly those with refined sugars, may be toxic to the human body and contribute to cancer progression. The consumption of fructose, a component of many sweetened foods, has been associated with an increased risk of intestinal tumours in pre-clinical [21]. Fructose levels are elevated in the bone marrow, and fructose facilitates tumor progression in patients with acute myeloid leukemia (AML) [22]. In parallel, non-nutritive sweeteners, such as saccharin, sucralose, aspartame, acesulfame-K, and stevia, have become a popular addition to the human diet because they do not contain calories. However, a randomized-controlled trial revealed that supplementing with sucralose and saccharin can impair glycemic response in healthy adults [23]. Additionally, studies on mice suggest that a high intake of sucralose can increase the risk of tumorigenesis in subcutaneous cancer models by dampening antigen-specific responses in CD8^+^ T cell [24]. Hence, the consumption of non-nutritive sweeteners should be thoroughly investigated to determine if it is associated with adverse health effects and cancer risk. In contrast, dietary fibers, which are nondigestible carbohydrates, have been associated with reduction of overall cancer risk. This may be due to their beneficial effects on the intestinal microbiota (see below).

Low-protein diets have rarely been used in the treatment of cancer patients. However, epidemiologic studies have linked cancer risk to the consumption of animal protein rather than plant-based protein [25]. In mouse models, a low-protein diet has been found to decrease tumor growth by triggering the IRE-1α-mediated endoplasmic reticulum stress response. This leads to the production of immunostimulatory cytokines, expansion of CD8^+^ T cells and antigen-presenting cells (APCs) [26].

It is essential to note that the alterations being discussed affect several food components, including the absorption of dietary fibers, complex carbohydrates, and polyphenols present in plant-based foods. Consequently, it is challenging to draw a definite conclusion that a reduced consumption of essential amino acid is solely responsible for these effects, which be further discussed to shed more light on the matter. In conclusion, shifting macronutrients in an isocaloric manner has demonstrated the anticancer effects. This hypothesis is currently under evaluation in various clinical trials. However, it is essential to conduct further research to determine the complete benefits and risks of particular dietary modifications on cancer progression.

### Interventions on micronutrients

Micronutrients are essential for human metabolism and neoplastic disease. They contain mainly vitamins and minerals, but also essential amino acids and essential fatty acids [27]. Cancer cells have the ability to alter their metabolism in response to varying metabolic environments, and each oncogenic pathway is associated with unique modifications in biochemical pathways, leading to specific metabolic dependencies. This presents an exciting opportunity for precision oncology, where specific micronutrients can be removed from the body to target metabolic vulnerabilities in cancer cells while preserving the functionality of anti-tumorous cell types, including cytotoxic T lymphocytes and NK cells that are vital for restricting malignant growth.

#### Vitamins

Vitamins are organic molecules that are essential to an organism in small quantities for proper metabolic function, including Vitamin A, B, C, D, E, K. Although multitudinous clinical trials have explored the supplementation of various vitamins and oligoelements for a wide range of malignancies, most have yielded disappointing consequences. Despite this, patients frequently use polyvitamin pills and antioxidant supplements, which have been shown to have negative effects, such as increasing the recurrence rate of breast cancer treated with chemotherapy [28]. Additionally, clinical trials, such as the SU.VI.MAX trial involved over 12,000 patients, showed no evidence of a decrease in overall cancer incidence with antioxidant supplementation [29]. Consequently, patients should avoid taking such supplements unless instructed otherwise. Nevertheless, there is compelling evidence supporting the use of certain micronutrients for preventing and treating certain types of cancer.

Consuming vitamin A (retinal, retinoic and retinol acid) and its provitamins, especially β-carotene, can moderately lower the risk of certain cancers, such as non-small-cell lung cancer (NSCLC) [30] and pancreatic cancer (PDAC) [31]. Meanwhile, pharmacological doses of all-*trans*-retinoic acid (ATRA) are highly effective in inducing differentiation in specific leukemia cell types and are frequently used to treat promyelocytic leukemia and AML [32].

Niacin and niacinamide, commonly known as vitamin B3, are essential components for the production of nicotinamide-adenine dinucleotide (NAD^+^). NAD^+^ is a crucial substrate and cofactor for several enzymes, including poly ADP-ribose polymerase 1 (PARP1) and SIRT1. Consuming high amounts of vitamin B3 has been linked to a reduced risk of developing hepatocellular carcinoma in humans [33]. Furthermore, studies have shown that niacinamide (NAM) has a synergistic effect when combined with gemcitabine against PDAC [34], and niacin exhibits favorable interactions with temozolomide against glioblastoma [35]. The strongest evidence from phase 3 trials suggests that nicotinamide can be used prophylactically to prevent nonmelanoma skin cancer [36], and can also serve as an adjunct to radiotherapy in the treatment of bladder and laryngeal cancers [37,38]. Notably, supplementing with nicotinamide has been found to have chemopreventive effects against breast cancer with luminal B subtype in mice, and it can increase the effectiveness of anthracycline-based chemotherapy for breast cancer and fibrosarcoma [39]. These findings underscore the broad anticancer potential of vitamin B3.

Vitamin C, also known as ascorbate, has been proposed to have cancer-preventive effects, but the results have largely been disappointing, with the exception of higher dietary vitamin C intake being linked to a lower risk of lung cancer [40]. Recently, vitamin C is found to directly modifies lysine residues through vitcylation, regulating STAT1 signaling and enhancing antitumor immune responses by preventing STAT1 dephosphorylation [41]. However, in a phase I trial, administering high doses of ascorbate intravenously has been found to potentiate radiotherapy, leading to improved progression-free survival for pancreatic cancer patients [42]. A high-dose solution containing 30 % ascorbic acid is more efficient than imiquimod for the treatment of basal cell carcinoma in a randomized trial[43]. It is worth noting that ongoing clinical trials should focus on the intravenous supplement of vitamin C for cancer treatment.

Vitamin D has been found to cause a slightly reduction in the risk of cancer mortality, although it did not reduce overall cancer incidence [44]. In a large randomized trial, supplementation of vitamin D merely diminish the risk of advanced cancer, while fails to reduce overall cancer incidence [45]. Studies have demonstrated a correlation between serum concentrations of 25-hydroxyvitamin D3 and the clinical response of patients with EGFR-mutant lung adenocarcinoma to EGFR inhibitors [46]. High circulating 25(OH)D3 levels are associated with optimal overall and progression-free survival in advanced colorectal cancer patients [47]. Additionally, in a randomized trial, vitamin D specifically lowers the incidence of intraductal breast cancer in situ [48].

In contrast to the established roles of supplementation, recent studies have revealed that the targeted restriction of certain vitamins may also confer antitumor benefits by interfering with tumor-specific metabolic dependencies. Recent studies demonstrate that dietary restriction of vitamin B5 can suppress MYC-driven tumor progression by limiting CoA biosynthesis and disrupting metabolic programs essential for cancer cell proliferation [49]. Similarly, excess dietary folate promotes hepatocellular carcinoma by stabilizing MAT2A, a key enzyme in methionine metabolism, whereas folate deprivation reverses this effect and delays tumor development [50]. In addition, high dietary intake of menaquinones (vitamin K2) has been associated with an increased risk of breast cancer incidence and mortality in a large prospective cohort study, suggesting that restricting vitamin K2 consumption may provide a potential preventive strategy in breast cancer management [51], suggesting that, in some contexts, restriction may provide a preventive strategy.

Despite this promising direction, vitamin-restricted strategies face significant translational hurdles. These include patient adherence to long-term restrictive diets, heterogeneous responses due to metabolic variability, and the absence of validated biomarkers to identify patients who would benefit from such interventions. Moreover, vitamin restriction may inadvertently impair normal tissue homeostasis, particularly in immune or proliferative cells. Therefore, future research should focus on optimizing dosing, identifying predictive biomarkers, and conducting carefully stratified clinical trials to refine and personalize dietary vitamin modulation strategies for cancer therapy.

#### Minerals

Dietary minerals, including phosphorus (P), potassium (K), chloride (Cl), copper (Cu), iodine (I), iron (Fe), zinc (Zn), magnesium (Mg), sodium (Na), selenium (Se), and calcium (Ca), exhibit bioactivity by chelating with various elements. This process plays a crucial role in physiological processes by modulating microbiome metabolism or antioxidant capacity. Beyond these classical roles, growing evidence indicates that dysregulated mineral metabolism is intimately involved in cancer development, progression, therapeutic response, and prognosis.

Given their roles in redox balance and immune surveillance, many minerals have garnered attention for their potential cancer-preventive properties. Zinc, for example, supports immune function and is inversely associated with esophageal squamous cell carcinoma risk, particularly in high-incidence areas. Selenium, known for its potent antioxidant and anti-inflammatory effects, exhibits a dose-dependent inverse association with the incidence of pancreatic and esophageal cancers. Moreover, elevated serum selenium levels are correlated with improved survival across several malignancies [52,53]. Additionally, selenium and zinc have been linked to a lower risk of colorectal and prostate cancers, potentially by reducing DNA damage and enhancing T-cell-mediated antitumor immunity [54]. Conversely, excessive copper accumulation has been associated with tumor progression, especially in colorectal cancer, possibly through promoting oxidative stress and inflammatory signaling [55,56].

In addition to prevention, abnormal mineral levels are increasingly recognized as diagnostic and prognostic biomarkers. Elevated cobalt and decreased selenium concentrations have been identified in the serum of pancreatic cancer patients, while higher copper-to-zinc ratios are linked to advanced colorectal cancer stages [57,58]. Moreover, dynamic changes in serum selenium and zinc levels during treatment are predictive of clinical outcomes, particularly in pancreatic cancer [58]. Beyond systemic circulation, trace elements may also shape the tumor microenvironment (TME). For example, zinc can activate M1 macrophages and CD8^+^ T cells, thereby enhancing responsiveness to immunotherapy [59], while dysregulated iron metabolism supports tumor proliferation and metastasis and is under investigation as a therapeutic target [49].

Minerals also exert direct anticancer effects or enhance therapy through mechanisms such as ferroptosis induction, apoptosis, and immune activation. Selenium, copper, arsenic, and manganese have been shown to suppress tumor growth and restrain metastatic spread in various models [60]. Copper may also mediate “cuproptosis”, a novel form of cell death with therapeutic relevance in cervical and other malignancies [56]. Zinc supplementation has demonstrated protective effects in renal cancer, while higher serum magnesium has been associated with prostate cancer risk [61]. Importantly, zinc and manganese have also been implicated in modulating immune checkpoint expression and T cell activation, improving responses to immunotherapies [61,62]. Selenium has been reported to alleviate the immunosuppressive side effects of chemotherapy and radiotherapy, potentially improving patient quality of life [54]. In addition, selenium has been shown to stimulate antitumor immunity by reversing immunosuppression in the tumor microenvironment and promoting the activation of immune effector cells such as M1 macrophages and CD8^+^ T cells [63].

Nutritional interventions involving trace elements are gaining traction as adjuncts to conventional therapies. High-dose multimineral supplementation has been suggested to potentiate chemotherapy and radiotherapy while reducing toxicity [64]. Conversely, deficiencies in key minerals such as selenium and zinc may impair immune responses and reduce therapeutic efficacy [65]. These insights underscore the need for personalized nutrition strategies, especially for patients undergoing intensive cancer therapy. Moreover, mineral levels influence gut microbiome composition, which in turn affects systemic immunity. For example, Bifidobacterium species can sequester free iron to reduce colorectal tumorigenesis, while zinc and selenium modulate mucosal immune cell activity [55]. Probiotic and prebiotic strategies that alter trace element availability in the gut are also being explored to synergize with cancer immunotherapy [66].

Importantly, the effects of trace elements are highly context-dependent and vary with cancer type and genetic background. For instance, manganese levels negatively correlate with KRAS mutations in colorectal cancer, rubidium levels are associated with microsatellite instability (MSI), and Mendelian randomization supports a protective role for zinc and a detrimental effect of magnesium in prostate cancer [[61], [67], [68], [69]]. Heavy metals such as lead, cadmium, and arsenic are consistently associated with increased cancer risk across organ systems [67].

Despite growing interest, the translation of mineral-based interventions into clinical oncology remains limited by several challenges. These include tumor heterogeneity, environmental exposures, variability in patient genetics, and the narrow therapeutic margin between deficiency and toxicity. Moving forward, more rigorous mechanistic studies and biomarker-guided trials are needed to define causality and guide the rational use of trace elements in precision cancer prevention and therapy.

#### Amino acids

Amino acids play a crucial role in the growth and development of organisms, and are classified as 'essential' or 'nonessential' depending on their dietary requirements. However, cancer cells unapplied the classification of amino acids. Cancer cells often require 'non-essential' amino acids for anabolic processes, and depriving them of specific amino acids can significantly compromise their fitness, even if they are capable of synthesizing those amino acids themselves.

To illustrate, cancer cells necessitate abundant methionine levels to sustain their growth [70]. Methionine is eager during translated process and serves as a direct donator for S-adenosylmethionine (SAM) to mediate methylation reactions including histone and DNA methylation in a SAM-dependent method [71]. The potential of dietary methionine restriction to enhance cancer treatment was first suggested in the early 1990s, and has been demonstrated in various mouse models and types of cancer [72]. For instance, it has been reported that dietary methionine restriction could promote cyclic GMP-AMP synthase (cGAS) activation through reversible methylation and enhance cGAS-mediated antitumor immunity [73]. One week of methionine-restricting diet is enough to produce remarkable metabolic remodels in mice [74]. Recent studies have also found that humans who limit their methionine intake experience similar metabolic alterations to those observed in methionine-limited tumor-bearing mice, a phenomenon exhibiting a suppression of nucleotide synthesis and one-carbon metabolism [72]. These promising findings suggest that restricting methionine in cancer patients is likely to have a similar anti-tumorigenic effect as observed in mouse models. Interestingly, plant-based vegan diets, which exclude animal proteins, are naturally low in methionine, suggesting a biochemical explanation for their reputed health benefits. In clinical, patients with metastatic prostate cancer were orally administered methioninase, which resulted in a decrease in their PSA levels [75]. It's uncertain whether the benefits of methioninase will translate to improved survival on a clinical level. However, it's important to note that methionine deprivation comes with drawbacks. For instance, limiting both cysteine and methionine specifically stimulate endothelial cells to release vascular endothelial growth factor (VEGF), which has been shown to have a pro-tumor effect. This should be taken into account when suggesting dietary changes to cancer patients [76]. In contrast, recent study demonstrates that a diet pattern with cysteine depletion and methionine restriction is capable to sensitize neoplastic cells to ferroptosis and enhance the efficiency of ferroptotic inducer to prolong survival [77]. Consequently, research indicates that long-term methionine restriction is a safe and advantageous dietary strategy to prevent cancer in healthy individuals.

Discussing amino acids restriction in diet, muscle atrophy is capacity to augment the levels of all non-deprived amino acids in the circulation [78]. Consequently, even if dietary protein is completely removed, it may still be ineffective in decreasing the levels of most amino acids in the bloodstream [79]. Thus, depleting amino acid levels in the tumor microecosystem without causing a severe systemic reduction of these amino acids, which could be toxic, is not trivial. From a technical standpoint, humans can achieve the restriction of specific amino acid through defined protein beverages supplemented with vegetables, fruits, and grains. While studies have demonstrated the cancer-restraining functions of specific amino acid depletion in mice, translating this into clinical trials is difficult due to the logistics of preparing close-to-synthetic diets. However, administering enzymes that selectively eliminate specific amino acids may be a feasible alternative for cancer treatment, although this is typically beyond the scope of dietotherapies [80].

#### Unsaturated fatty acids

Research suggests that high concentrations of omega-3 PUFAs (eicosapentaenoic, docosapentaenoic, and docosahexaenoic acids) in the bloodstream are linked to lower overall and cancer-related mortality [81]. Immunonutrition employs specialized substrates termed immunonutrients to modulate immune and inflammatory pathways [82]. The most extensively studied include omega-3 fatty acids, glutamine, sulfur-containing amino acids, antioxidants, arginine, and nucleotides, used individually or in combination [83]. Omega-3 PUFAs as immunonutrition enhance immune responses and potentially prevent cancer. A high intake of omega-3 PUFAs has been linked to a reduced risk of colorectal cancer in individuals with a high level of FOXP3^+^ T-cell density, suggesting omega-3 PUFAs have capacity for cancer immunoprevention by mediating regulatory T cells [62]. Fish-based unsaturated fat, commonly found in the Mediterranean diet, may be particularly beneficial. Studies have shown that patients on this diet have better sensitivity to treatment with immune checkpoint blockade and improved progression-free survival [11]. Ongoing trials are investigating the potential of omega-3 PUFAs alone or in combination with additional nutrients to improve the outcomes of cancer treatments are under way (Table 2).

Ferroptosis is a type of programmed cell death that depends on iron and occurs as a result of uncontrolled lipid peroxidation, which eventually leads to the rupture of the plasma membrane. Ferroptosis can be triggered by either intrinsic or extrinsic pathways [84]. The extrinsic pathway is triggered by the activation of iron transporters such as lactotransferrin and serotransferrin, or by the inhibition of cell membrane transporters, including the cystine/glutamate transporter. In contrast, the intrinsic pathway is initiated by blocking cellular antioxidant enzymes like glutathione peroxidases [85]. Ferroptosis has a multifaceted role in tumorigenesis and tumor therapy, which is influenced not only by tumor suppressors and oncogenes but also by the tumor ecosystem. For example, 7-dehydrocholesterol (7-DHC) is recently identified as a natural anti-ferroptotic metabolite [86,87]. Both omega-3 and omega-6 polyunsaturated fatty acids (PUFAs) can preferentially accumulate in tumor cells under ambient acidosis conditions to peculiarly stimulate ferroptosis. Additionally, fish-based diet is capable to elevate a circulating level of omega-3 PUFA to significantly retard mouse tumor growth [88]. In the tumor microenvironment, pathologically activated neutrophils (PMNs) undergo spontaneous ferroptotic cell death. Although this reduces the presence of PMN-myeloid-derived suppressor cell (MDSCs), ferroptotic neutrophils enable to secrete oxygenated lipids to damage the function of effector T cell. The modulation of ferroptotic pathways has been implicated in various cancer treatment strategies such as radiotherapy, chemotherapy and immunotherapy. It is crucial to consider the interplay between ferroptosis and immunity when developing new therapeutic approaches.

In summary, there is fragmentary evidence indicating that supplementing specific micronutrients like specific vitamins in preclinical models enhance the effectiveness of cancer treatments. Clinical studies suggest that oral nicotinamide, topical ascorbate, and vitamin D supplementation could be beneficial for preventing or treating certain types of cancer. Ongoing and future trials are essential in determining the clinical efficacy of these micronutrient interventions for neoplastic disease prevention and treatment (Table 2). Finally, the appliance of comprehensive mass-spectrometric metabolomics could provide valuable insights into identifying novel metabolites associated with complete therapeutic responses or favorable cancer prognosis.

### Microbial diet

Approaches that include nutritional interventions for cancer therapy necessitate to consider their potential impact on gut microbial diversity, which potentially modulating cancer progression and the response to cancer therapy [[89], [90], [91]]. Clinical trials have shown that fecal microbial transplantation can enhance the efficiency of immunotherapy [92,93], while there is an extensive consensus that the administration of antibiotics especially macrolide during ICI treatment has been found to detrimentally influence therapeutic effects [94,95]. Hence, these studies underscore the indispensable role of gut microbiota in determining the response to cancer therapy. The effects of the gut microbiota on cancer progression mainly impacts tumorous or systemic metabolic state and immunologic status via multifarious effectors including the generated metabolites (vitamins, tryptophan catabolites and polyamines) and elicitation of specific immune reactions [[96], [97], [98]]. In fact, diverse diet can have a strong influence on the composition of the gut microbiome. For example, the supplementation of dietary fiber like arabinoxylan and long-chain inulin (LCI) has been found to reduce cholesterol levels, and LCI is attributable to enrichment in *Bifidobacterium,* which is associated with a decreased cancer risk [99]. Interestingly, ketogenic diet has also been found to increase the relative abundance of *Akkermansia muciniphila* [100], a bacterium that can reconstruct the response to immune checkpoint blockade in cancer patients and tumor-bearing mice [91,93]. Here, we summarize four classifications of microbial diets, including prebiotic, probiotic, synbiotic (containing pre- and probiotics) and postbiotic diets.

Prebiotics are compounds that are indigestible by human enzymes or unabsorbed by the intestine, and benefit the activity and growth of gut microbiota. The emblematic prebiotic is dietary fiber, which can be degraded and fermented into short chain fatty acids (SCFAs) through certain beneficial bacteria like *Lactobacilli spp*, *Ruminococcaceae, Lachnospiracea*e, as well as *Bifidobacteriaceae* [101]. SCFAs are discovered to reinforce cellular and humoral immune responses to immunotherapy [102]. Recent studies have suggested that fiber-enriched diet unaccompanied by probiotic products are contributing to improve progression-free survival in advanced melanoma patients treated with immunotherapy [103]. However, it is confused whether over-the-counter probiotics and dietary fibers independently influence the efficiency of immunotherapy, calling for ongoing trials (NCT04645680). Several trials are also underway to evaluate the tumor-suppressive potential of chemically defined fibers or oligo-fucoidan (sulfated polysaccharides) (Table 2).

Probiotics are live microorganisms that are orally administered with the intention of colonizing the intestinal microbiota. Probiotics partially contain *Clostridium butyricum*, *Lachnospiraceae*, *Faecalibacterium* species (*Ruminococcaceae* family members), *Enterococcus faecium*, *Bifidobacterium longum*, *Lactobacillus*, *Collinsella aerofaciens*, and *Akkermansia muciniphila*, administered to patients through FMT or commercial formulations. *Clostridium butyricum* is an anaerobic, spore-forming bacterium that produces butyrate. *Clostridium butyricum* therapy circumvents antibiotics-induced dysbiosis and has a positive impact on therapeutic efficacy of ICB in patients with non-small cell lung cancer [104]. Similarly, supplementation with CBM588, a bacterial product containing live *Clostridium butyricum*, augment the clinical efficacy of ICI and improve the clinical outcome in patients with metastatic kidney cancer [105]. In addition, an orally administered microbial consortium, known as Microbial Ecosystem Therapeutic 4 (MET4), consisting of 30 cultivated species, is being used as an alternative to FMT in advanced cancer patients receiving ICI treatment. The results of this study support the further development of microbial consortia as a therapeutic co-intervention for ICI treatment in cancer patients [106].

Postbiotics are metabolites, active peptides and nucleic acid substances secreted by the microbiota to directly or indirectly benefit on their host. Most obviously, the microbiota can synthesize several nutrients such as fatty acids, and provide these to the host. In contrast, microbiota have the ability to consume preferential nutrients and thereby affect their availability within the body. For example, gut microbiota is capable to convert some specific amino acids including methionine, arginine and tryptophan. Recent study has shown that gut microbiota enables to generate indole-3-acetic acid (3-IAA) by catabolizing tryptophan, and manipulating tryptophan intake in the short-term potentiate chemotherapy efficacy in humanized gnotobiotic mice bearing PDAC. Mechanically, 3-IAA downregulates glutathione peroxidase 3 (GPX3) and GPX7, two reactive oxygen species (ROS)-degrading enzymes. Subsequently, ROS is accumulated in cancer cells to retard tumor proliferation [97]. In similar, *Lactobacillus reuteri* enable to decompose tryptophan into indole-3-aldehyde (I3A), which drives effector function of T cells via inducing aryl hydrocarbon receptor-dependent CREB activity. *Lactobacillus reuteri* supplement, dietary tryptophan or I3A administration enhance ICI response and survival in melanoma patients [96]. Finally, active peptides, ornithine lipids and diacyl phosphatidylethanolamine generated by *Akkermansia muciniphila* induces homeostatic immune responses and improve host metabolic homeostasis [[107], [108], [109], [110]].

Overall, these observations suggest that specific microbial diets, including probiotics and postbiotics, have the potential to modulate cancer progression and the response to therapy, and warrant further investigation in future studies.

## Multiscale mechanisms: From molecular sensors to ecosystem remodeling

**Fig. 2:**
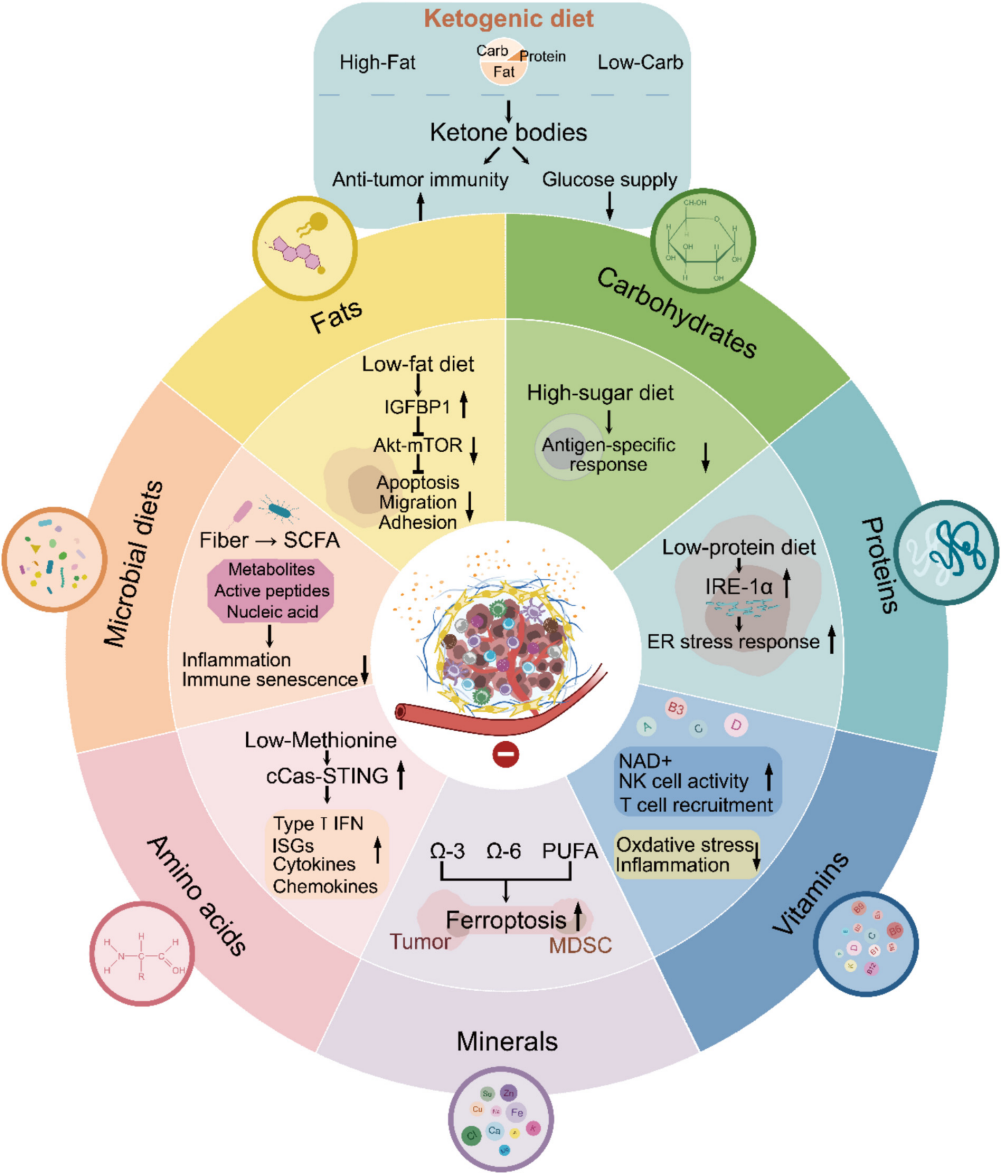
Mechanisms through which dietary components influence tumor progression. This figure summarizes how dietary interventions modulate tumor progression via specific mechanisms. Macronutrients like carbohydrates, fats, and proteins affect cancer development through oxidative stress, inflammation, and immune modulation. For instance, ketogenic diets reduce glucose levels and enhance anti-tumor immunity, while low-protein and low-fat diets regulate pathways like ER stress and Akt-mTOR. Micronutrients, including vitamins (e.g., A, D, E, B-complex) and minerals (e.g., Mg, Zn, Se), modulate oxidative stress and immune responses. Microbial diets, such as fiber-derived SCFAs, enhance antigen-specific immunity, and omega-3 fatty acids promote T cell recruitment and NK cell function. These dietary components collectively target tumor progression and immune regulation, offering insights for nutrition-based cancer therapies.

Interest is growing in more targeted methods to nutritive interventions for cancer therapy, which involves selectively removing specific nutrients depending on the definite mechanisms of the tumor and systemic requirements (Fig. 2). This approach necessitates stratification of tumor ecosystem, and can be termed “precision nutrigeroscience”. The metabolic changes in the tumor ecosystem, modulated by dietary nutrients, are diverse and plastic, and affect cancer progression in distinct ways.

Precision dietotherapies will be customized to address not only the metabolism of cancer cells themselves but also the broad ecological environment including microenvironment (the crosstalk between stromal/immune cells and tumor cells) and macroenvironment (neuroendocrine modulation, immunological response, systemically metabolic states and gut microbiota homeostasis). To make informed therapeutic decisions, it is crucial to have a comprehensive understanding of how these various factors intersect. Recent advancements in research have led to exciting progress in identifying these interactions, and a clear mechanistic understanding of them is necessary for success in precision diet therapies.

### The cellular nutrient sensors

The cellular mechanisms responsible for sensing nutrimental alterations are generally considered universal. Several nutrient sensors have been identified, such as the acetyltransferase EP300, protein kinase mechanistic target of rapamycin complex 1 (mTORC1), and others that detect nutrient scarcity, such as the deacetylases (SIRT1 and SIRT3) and AMP-activated kinase (AMPK). These sensors enforce catabolism (lipolysis coupled to ketogenesis, glycogenolysis, gluconeogenesis and proteolysis) and stimulate adaptive cellular stress responses (including antioxidant reactions, autophagy, and DNA damage repair) and epigenetic alterations [5].

One of the primary molecular mechanisms related to nutrient sensing is the mammalian target of rapamycin (mTOR) kinase, existing in two forms: mTORC1 and mTORC2. Both separate complexes are composed of multitudinous protein subunits function to phosphorylate diverse substrates, and serve different functions. Specifically, mTORC1 plays a crucial role in controlling various external stimuli, such as amino acids, glucose, oxygen availability, insulin/insulin-like growth factor 1 (IGF-1), and cholesterol. Moreover, mTORC1 integrates various extracellular and intracellular nutrient signals, which involve macromolecule biogenesis as well as protein translation and degradation [111]. By inhibiting mTOR signaling, dietary restriction delays age-related pathologies in humans. Calorie restriction exemplifies this effect, promoting longevity, improving cognitive performance, strengthening cardiac health, ameliorating metabolic disorders, and lowering cancer risk [112].

In addition, AMPK functions as a detector of mitochondrial stress and energy status, and can be activated when the AMP:ATP ratio is elevated. Functionally, this activation triggers several catabolic pathways, including fatty acid oxidation and glycolysis, as a timely response to low levels of cellular energy, ultimately supplementing ATP levels [113]. In *C. elegans*, glucose restriction triggers mitohormesis, characterized by elevated reactive oxygen species (ROS) and activation of oxidative stress resistance. This glucose restriction-induced response, dependent on AMPK, is essential for extending lifespan [114].

Moreover, Sirtuins require NAD^+^ for their catalytic functions, including desuccinylation, deacylation, depalmitoylation, demalonylation, demyristoylation, and mono-ADP-ribosylation, thereby connecting diet with metabolic processes. NAD^+^ acts as a master coenzyme in antioxidant defense, and several non-redox enzymes like sirtuins and poly-ADP-ribose polymerases (PARPs) require NAD^+^ as a cofactor [115].

Dietary limitation of total protein or key amino acids (branched-chain amino acids, methionine, cysteine, glutamine) stimulates autophagy/lysosomal activity, mediated partly by mTOR suppression and GCN2 kinase activation [116]. As an evolutionarily conserved serine/threonine kinase, GCN2 has capacity to sense the changed levels of amino acid to initiate various downstream metabolism-response pathways. For instance, GCN2 enables to modulate immune homoeostasis and malignant growth through coordination with inflammation and integrated stress responses. GCN2 can be activated by uncharged tRNAs elevation and ribosome stalling to phosphorylate eukaryotic translation initiation factor 2α (eIF2α), then blocking major translation of most mRNAs but selectively stimulating ATF4-mediated translation [117]. Additionally, upon valine deprivation, human histone deacetylase 6 (HDAC6) accumulates in the nucleus and causes DNA damage. Mechanistically, nuclear-localized HDAC6 deacetylates ten-eleven translocation 2 (TET2) to activate DNA demethylation, which subsequently induces DNA damage through excision mediated by thymine DNA glycosylase [118].

While the primary modulators of cellular nutrient-sensitive mechanisms are explicitly established, future studies could focus on identifying and validating generalized dietetic effector mechanisms to develop individualized nutritious regimens for cancer treatment.

### The tumor microenvironment

It is widely recognized that tumor development and therapeutic response are heavily influenced by the tumor microenvironment and local nutrient availability [10,119]. The metabolites availability intrinsically varies among distinct tissues, which can impact the efficacy of dietary therapies in different ways. In organs with abundant nutrients, limiting dietary intake alone may not sufficiently reduce essential metabolites to impede tumorigenesis. Conversely, tumors originating in nutrient-depleted organs or regions may be more susceptible to dietary restrictions. In order to satisfy their nutrient requirements, cancerous cells have been observed to scavenge lipids, polypeptides, and even proteins from the extracellular matrix, interstitial fluid and stromal cells using various approaches such as micropinocytosis, entosis, endocytosis, or exosomes uptake exosomes [120,121]. Stromal cells surrounding the cancer can provide several necessary nutrients for tumor growth through the induction of autophagy [122]. Moreover, tumor cells energetically remodel the biological activities of the neighboring stromal cells to boost neoplastic growth and survival. It has been observed that parathyroid-hormone-related protein (PTHrP) secreted by renal cancer cells stimulate the browning of perinephric adipose tissue, in turn, generating excessive lactate to enhance tumor growth and metastasis [123]. Within TME, stromal cells mainly contain adipocytes, endotheliocyte and fibroblasts. Tumor cells depend heavily on these cells for mechanical or trophic sustentation via adipolysis, angiogenesis and fibrational support. For instance, cancer-associated adipocytes or fibroblasts are capable to release provide various nutrient substances to the tumor [123,124]. Although these types of cancers may be suitable candidates for nutrient-synthetic inhibitors, it’s potential that certain tumors adapted to sparseness of external nutrients may be resistant to the therapies of metabolic interventions. Finally, the intratumor microbiota has been identified as an integral tumor component, but the paratrophy between intratumor microbiota and malignant cell still remains unknown [125].

### The tumor macroenvironment

The concept of tumor macroenvironment transcends the tumor-intrinsic characterization and the tumor microenvironment. The continuous interaction between the host and the tumor via the lymphatic, vascular, and nervous systems can lead to long-distance effects. The crosstalk is modulated by neuroendocrine factors, metabolites derived from gut microbiota or host, tissues hormones, immunostimulants and immunosuppressants. These modulators represent the paramount aspects of tumor macroenvironment with respect to tumorigenesis and cancer development. The tumor macroenvironment can be stratified into four systemically distinct but coadjutant layers, termed (1) neuroendocrine circuitries, (2) systemic immunity, (3) systemic metabolism, and (4) microbiota. The importance of diet-mediated macroenvironment for malignant progression will be discussed using preclinical and clinical evidence.

#### Neuroendocrine circuitries

Neuroendocrine circuitries play a crucial role in cancer development, with variations in nutrimental support affecting the circulating levels of protein hormones (like Fibroblast growth factor 21 (FGF21), insulin/IGF1 or leptin), neurotransmitters or neuropeptides (such as catecholamines), and steroids (like glucocorticoids). These molecules have an impact on the biologic behavior of cancer through mediating the cancer-host interplay or eutrophic support. First of all, excessive long-term calorie intake raise in IGF1 levels, which has been found to be associated with cancer risk, as pharmacologically blocking IGF1R by linsitinib or picropodophyllin is able to ameliorate cancer-targeting immunosurveillance by stimulating autophagic immunostimulants in cancer cells [126]. Subsequently, neuropeptides are discovered to reshape considerable facets in tumorous biology, potentially connecting neural activity with oncogenesis. Notedly, acyl-coenzyme A binding protein (ACBP), an endogenous allosteric activator of the GABAA receptor and an appetite stimulants, is secreted by hepatocyte under hunger or obese state [127]. The ACBP-mediated homeostasis is inordinate in patients with advanced cancer [128]. In mechanism, ACBP accelerates malignant growth by availably transporting long-chain fatty acyl-CoAs to mitochondria and promoting fatty acid oxidation [129]. These examples exemplify the possibility that ACBP can be a potential therapeutic strategy for proliferative cancer. In addition, nutrient-sensing neurons named agouti-related peptide (AgRP) neurons induce autophagy and promote ketogenesis after fasting. The activation of AgRP neurons release neuropeptide (NPY) to presynapticly inhibit NPY1R-expressing neurons in the paraventricular nucleus of the hypothalamus (PVH), subsequently stimulate PVH^CRH^ neurons (corticotropin-releasing hormone, CRH) to secrete corticotropin-releasing hormone (CRH). Furthermore, the activation of AgRP neurons increases the levels of circulating corticosterone induces hepatic autophagy in a glucocorticoid receptor (GR)-dependent way [130]. Ultimately, the immunosuppressive functionality of glucocorticoids intensively impacts anticancer immunosurveillance. Dietary restriction induces to an increased level of circulating glucocorticoids but sustain a low level of glucocorticoids in bone marrow, that contributes to recruit and maintain memory T cell in bone marrow [131]. In mice, glucocorticoids administration has capacity to impair cancer immunosurveillance through TSC22D3 activation in dendritic cells. Close correlations have been found between circulating cortisol levels, depression-anxious mood, TSC22D3 expression in circulatory leukocytes, and worse prognosis in patients with certain types of cancer. Mifepristone, a GR antagonist, is administrated to enhance anti-tumor immunosurveillance in stressful mice [132]. Therefore, the ongoing debate regarding the extensive application of corticosteroids for preventing chemotherapy-evocated vomiting and nausea raises questions about their effectiveness and safety.

#### Systemic immunity

For cancer cells to thrive, they must evade immunosurveillance, which can be accomplished through systemically or locally immunosuppressive circumstance. Immunometabolism is susceptible by fluctuations in nutrimental supplement to reinforce or weaken cancer immunosurveillance. The metabolic features of cancer cells must be discrepant to those in adjacent normal tissue. However, it is potential that cancer cells adopt metabolic status under normal physiological circumstances, such as during the repair process of damaged tissue, speedy expansion of effector T cell, or stemness maintenance [133]. To achieve the desired outcome of dietary interventions aimed at targeting tumor metabolism by altering systemic nutrient availability, it is essential to recognize that these interventions may also affect the behavior of normal tissues. Consequently, such effects could have significant implications for the success of these interventions. Immunological recognition and immune elimination that plays a critical role in regulating tumor progression are illustrated for the confounding effect on cancer treatment. Indeed, several efficient immunotherapies have been established to reshape functionality of tumor-suppressive lymphocyte. Given the connection between immune homeostasis and nutritional status, nutritional interventions designed to retard neoplastic growth may have collateral effects [5,134]. In pre-clinical model, short-term fasting has been shown to improve anticancer immunosurveillance in preclinical models by depleting regulatory T cells (Tregs) in mice [135]. In clinical trial, FMD is capable to decrease the peripheral level of myeloid-derived immunosuppressive cells and the intratumoral infiltration of regulatory T cell (Treg), leading to enhanced intratumor T cells cytotoxicity and IFNγ enrichment to restore anticancer immunological surveillance [12]. In parallel, the tumor-reactive immune response has been designed to be enhanced by the ketogenic diet [136,137]. The interaction between the tumor-supportive immune reactions and the ketogenic diet deserves more focuses to evaluate its potential as a cancer therapy. As a key shared metabolic outcome of these interventions, beta-hydroxybutyrate (BHB), induced by fasting and ketogenic diets, plays a critical role in regulating immune cell development and function. It serves not only as a metabolic substrate to support the activity of CD8^+^ T cells and CAR-T cells, but also enhances their effector functions and memory formation through promoting TCA cycle activity and histone acetylation, highlighting its potential for clinical application in cancer immunotherapy [138,139]. Beyond host-derived metabolites like BHB, metabolites derived from the gut microbiome play important roles in systemic immunity. Among these, butyrate—a microbial short-chain fatty acid produced through fiber fermentation—has been shown to enhance antitumor immunity primarily by modulating cytotoxic CD8^+^ T cell function. Butyrate promotes IL-12 signaling and ID2 expression, which support CD8^+^ T cell effector differentiation and cytokine production. Moreover, butyrate epigenetically regulates T cell receptor (TCR) signaling through increased histone H3K27 acetylation, thereby enhancing responsiveness to immune checkpoint blockade and improving the efficacy of anticancer therapies, including chemotherapy and adoptive cell transfer [[140], [141], [142]]. Additionally, SLC13A3-mediated itaconate uptake promotes tumor immune evasion by alkylation-mediated stabilization of PD-L1, and targeting SLC13A3 enhances the efficacy of anti-CTLA-4 therapy in cancer [143]. Furthermore, specific amino acids restricted in nutrition strategies will have a favorable influence on anti-tumorous immune responses. Recent study shows that the effect of methionine-limited diet sensitizes tumor to PD-1 antibody treatment [73]. Detailedly, blocking methylation by methionine deprivation acts as a switch to release cGAS from chromatin and promote cGAS activity [73]. Hence, nutritional therapies are proposed to reinforce the anticancer immune.

#### Systemic metabolism

The role of systemic, bodywide metabolism in carcinogenesis is well-established. The risk of developing malignant disease can be affected by both the quantity, quality and composition of nutrients. Excessive nutrimental substances (glucose, lipids, amino acids) and bioplastic factors (such as insulin/IGF1) compel neoplastic cells to intake abundant nutrients, leading to a reduction of cytotoxic T cells-induced immunosurveillance [144]. In addition, red-meat consumption is a potential contributor for the inflammation-modulated colon carcinogenesis through mechanisms that an increase in *Clostridiales* and *Bacteroidales* to generate N-glycolylneuraminic acid (Neu5Gc), a product of heme iron or nitrates and nitrites to deteriorate DNA damage [145]. Conversely, recent study indicates that consuming coffee has an improved effect on risk of hepatocellular carcinoma, which is likely due to its ability to stimulate tumor-suppressive autophagy [146]. However, there are limited epidemiological associations that elaborate the effectiveness of individual dietary patterns on tumor risk and cancer development. Notedly, tumor cells have potential to impact the systemically metabolic sate, even in the early tumor stage. For example, inflammatory characteristics and disorder of gut microbiota are discovered in the ileum influence by cancer cells in an adrenergic stress-dependent way [147]. At later stages, tumor-associated cachexia exhibits the whole-body metabolic dysfunction driven by tumor-originating inflammatory factors and pro-cachectic cytokines [148]. This demonstrates how cancer and whole-body mechanisms is capable to mutually affect.

#### Gut microbiota

Gut microbiota exerts wide-ranging effects on systemic metabolism, immunoregulation, and immune cell repertoires to affect cancer development in distant, non-gastrointestinal cancers. The equilibration of gut microbiota is essential for maintaining the integrity of the intestinal barrier to preventing both systemic and local inflammation. The diversity and relative abundance of gut microbiota directly determines the inflammatory tonicity throughout the body [149]. As an example, fasting alters microbial taxa, including *Akkermansia, Hydrogenoanaerobacterium*, *Desulfovibrionaceae* and *Ruminococcaceae* to generate SCFAs and dampen local and systemic inflammation [150]. In similar, *Akkermansia muciniphila* produces a specific phospholipid acting as a hybrid agonist on the TLR1–TLR2 heterodimer [108], as well as an active polypeptide acting as a TLR2 agonist [109], that both have anti-inflammatory properties. Additionally, the gut microbiota provides several benefits for host immunity. For example, CR magnify overall diversity of gut microbiome to restore a favorable microbiome state, subsequently augmenting the components of naïve T cells/B cells to delay immunosenescence [151]. During early life, intestinal colonization results in microbial antigens recognition by intestinal dendritic cells, which hemotropically migrate into the thymus and stimulate rapid proliferation of microbiota-specific T cells [152]. Moreover, intestinal microbiota is found to present the cross-reactive antigens to stimulate the development of commensal-specific memory T cells. For example, the tail length tape measure protein (TMP), a cross-reacted antigens discovered in bacteriophage *Enterococcus hirae,* has been shown to improve immunotherapy efficiency by the intratumoral expansion of TMP-specific CD8^+^ T lymphocyte. Patients with high-expressed TMP-cross-reactive antigen in tumor or carrying enterococcal prophage in stools exhibit a long-period survival during treatment with PD-1 blocker [90]. Thus, the composition of gut microbiota is determined by one's lifestyle, and this, in turn, has an impact on the immune response against cancer.

## Clinical evidence and translational challenges of dietary interventions in cancer

Despite ongoing gaps in our mechanistic understanding of dietary interventions in the context of tumor control, robust preclinical findings have catalyzed a growing body of clinical research investigating their utility—particularly as adjuvant strategies designed to enhance the efficacy of standard therapies. Several randomized controlled trials (RCTs) and pilot studies evaluating dietary interventions in oncology are summarized in Table 2. Most of these approaches revolve around nutrient-restriction paradigms—such as CR, fasting, and glucose limitation—selected for their relative simplicity, feasibility, and ability to replicate some of the metabolic effects of fasting. In addition, other dietary strategies, including KD, amino acid restriction, Mediterranean diets, and micronutrient supplementation, are being explored in smaller, exploratory trials [153]. Although large-scale validation is lacking, early data have laid a critical foundation for the development of nutrition-based interventions in future clinical trials.

### Clinical trial evidence

To date, most clinical trials have prioritized the development of feasible and safe protocols for dietary interventions in patients with cancer. Implementing dietary regimens in oncology presents substantial challenges, as food choices are often deeply entrenched in individual preferences, cultural practices, and education. Consequently, strict dietary modification is inherently difficult, with dropout rates commonly approaching 30 %, even among motivated and well-informed participants [154,155]. To improve adherence, many trials incorporate supportive strategies such as preformulated meals provided by study centers, dietary counseling, and regular check-ins with clinical nutritionists. Despite these challenges, most studies have demonstrated that dietary interventions are generally safe and feasible. Common adverse effects—typically low-grade—include fatigue, gastrointestinal discomfort, transient hypoglycemia, and mild metabolic acidosis, reflecting adaptive responses to dietary changes. Severe adverse events are rare and generally restricted to older patients or those with significant comorbidities.

Short-term fasting and related interventions have been associated with improvements in several cancer-related metabolic markers, including reductions in adiposity, fasting glucose, insulin, IGF-1, and leptin levels. Studies have also reported successful induction of ketogenesis following fasting [156], FMD [157], and ketogenic dietary regimens [14,158,159]. Pilot trials combining fasting with chemotherapy have shown promising results in terms of quality of life, fatigue reduction, and attenuation of chemotherapy-associated toxicities; however, definitive evidence of enhanced therapeutic efficacy remains lacking.

Among these interventions, FMDs appear to have the most robust immunomodulatory effects, shifting systemic immune signatures toward phenotypes associated with improved clinical outcomes [160]. The DIRECT trial, a multicenter, open-label, randomized phase II study, evaluated the use of FMD cycles in combination with chemotherapy in patients with stage II–III HER2-negative breast cancer. While the trial did not demonstrate substantial clinical benefits in terms of pathological complete response or toxicity reduction, it did confirm that FMDs are well-tolerated and do not increase adverse event rates—even in the absence of prophylactic dexamethasone administration [161]. However, in the phase 2 trial BREAKFAST trial, a severely calorie-restricted, triweekly, 5-day FMD regimen results in excellent pathologic complete response (pCR) rates and long-term clinical outcomes when combined with preoperative chemotherapy in 30 patients with early-stage TNBC; and early downmodulation of intratumor glycolysis and pyruvate metabolism as a predictor of clinical benefit from nutrient restriction [162]. More recent case series have reported remarkable responses in patients with advanced-stage cancers receiving concurrent FMDs and standard therapy, including durable complete remissions in malignancies with otherwise poor prognoses [163].

Clinical studies investigating KD in cancer patients have primarily focused on early-phase assessments of safety and tolerability, with relatively few randomized controlled trials conducted to date [19,164,165]. Among them, the ERGO2 randomized pilot trial explored the combination of intermittent fasting and a calorie-restricted KD with reirradiation in patients with recurrent glioblastoma or gliosarcoma. The intervention led to pronounced reductions in blood glucose levels and successfully induced nutritional ketosis without eliciting diet-related adverse events. However, despite these favorable metabolic effects, the trial did not demonstrate significant improvements in progression-free or overall survival—likely attributable to the limited duration of the intervention [164,165]. In a separate open-label pilot study involving patients with advanced or metastatic breast cancer, a medium-chain triglyceride–based KD was evaluated for its safety profile, impact on body composition, and potential influence on survival outcomes. The study reported strong patient adherence, favorable physiological responses, and improved survival in the intervention group. Nonetheless, the generalizability of these findings is constrained by the small sample size and heterogeneity within the study population [19]. Expanding on the therapeutic potential of KD-inspired regimens, the CAPFISH-3 randomized clinical trial demonstrated that a high omega-3, low omega-6 dietary intervention—combined with long-term fish oil supplementation over one year—led to a significant reduction in the Ki-67 proliferation index, a biomarker linked to prostate cancer progression, metastasis, and mortality [166]. These findings underscore the potential of precision dietary modulation to influence tumor biology, although larger, more comprehensive trials are needed to validate clinical efficacy.

Currently, the majority of ongoing trials focus on fasting, FMDs, and CR regimens in cancers with limited treatment options and poor prognoses. This trend is especially evident in the context of glioblastoma and other primary brain tumors, where preliminary preclinical findings have highlighted the potential of ketogenic and fasting-based interventions to enhance therapeutic efficacy and prolong survival. Several trials—such as NCT01535911, NCT03278249, NCT05373381, and NCT03451799—are actively investigating these approaches in the clinic, with hopes of translating preclinical promise into improved patient outcomes. Other dietary strategies currently under investigation include restriction of nonessential amino acids as an adjuvant to chemotherapy in patients with metastatic pancreatic and colorectal cancers (NCT05078775 and NCT05183295), as well as modified Mediterranean diet protocols aimed at preventing breast cancer recurrence (NCT04174391).

Building upon the framework of precision nutrigeroscience, researchers are also working to identify rational diet–drug combinations that can enhance the efficacy of existing therapies and overcome resistance. One of the most promising pairings involves KD and phosphoinositide 3-kinase (PI3K) inhibitors. These inhibitors target PI3K pathway hyperactivation, a common metabolic hallmark in many human malignancies. However, their clinical utility has been limited by insulin feedback loops that drive resistance. Preclinical studies demonstrate that carbohydrate-restricted diets, such as KD, can suppress insulin feedback and thereby enhance the anti-tumor efficacy of PI3K inhibitors [167]. Several ongoing trials (NCT04750941, NCT05090358, NCT05183204) are currently evaluating KDs as adjuvant therapies in combination with PI3K inhibitors across a range of tumor types. The immunomodulatory potential of fasting and KDs has also prompted investigation into their use alongside immune checkpoint inhibitors. Given that many tumors exhibit primary or acquired resistance to checkpoint blockade, enhancing immune responsiveness through dietary intervention represents a compelling therapeutic avenue. Clinical trials (NCT05356182, NCT04387084) are now underway to assess whether metabolic reprogramming via diet can improve responses to these immunotherapies [4]. As cancer is inherently heterogeneous across tissue types, molecular subtypes, and host backgrounds, further efforts to delineate cancer–drug–diet synergies will be essential. Ultimately, individualized treatment protocols tailored to specific tumor contexts and patient profiles will be critical to maximizing therapeutic efficacy.

Although prior clinical studies have demonstrated the general safety and feasibility of dietary interventions in oncology, most cancer patients are still not provided with structured nutritional guidance during treatment. In fact, patients are often encouraged to increase caloric intake to avoid weight loss. While preclinical and early-phase clinical data suggest that nutritional modulation may improve outcomes, the clinical evidence remains inconsistent. Extracting reproducible and statistically meaningful results will require large-scale, rigorously controlled randomized trials that assess not only metabolic parameters but also survival and long-term disease progression in response to diet–therapy combinations.

The overarching goal of precision nutrigeroscience is to design patient-specific dietary regimens informed by tumor biology, immune status, metabolic phenotype, and treatment regimen, with the aim of maximizing therapeutic benefit while minimizing toxicity and adverse effects. To achieve clinical translation, standardized and reproducible dietary protocols must be established. This includes defining caloric targets, macronutrient ratios, micronutrient requirements, and permitted supplements. Equally important are the duration, frequency, and timing of dietary interventions, especially in relation to conventional therapies. Developing streamlined, evidence-based guidelines will be essential for the design and implementation of robust, multicenter clinical trials that can accelerate the integration of dietary therapeutics into standard oncologic practice.

### Translational challenges

As emphasized throughout this review, diet is a potent modulator of several hallmarks of cancer, including growth signaling, immune regulation, and microbiome dynamics. The study of dietary interventions in oncology is mechanistically nascent yet rapidly evolving. In the coming years, insights derived from clinical and preclinical studies are expected to deepen our understanding of cancer–nutrient interactions and inform the development of more sophisticated, evidence-based dietary strategies as adjuncts to conventional therapy.

Nonetheless, the translation of nutritional interventions into clinical oncology faces substantial barriers. Human dietary patterns are inherently variable, difficult to standardize or monitor with precision, and heavily influenced by cultural, psychological, and socioeconomic factors. This heterogeneity contributes significantly to inconsistent trial outcomes. Compounding these challenges are limitations in clinical trial design: many studies lack stratification based on critical host factors such as age, sex, microbiome composition, metabolic profile, and tumor subtype, thereby undermining the power of subgroup analyses. Adherence also remains a pervasive challenge, particularly with restrictive regimens such as CR or KD.

As the field moves toward precision implementation, ethical and safety considerations become increasingly urgent. Dietary interventions must be carefully tailored to avoid exacerbating malnutrition, especially in already vulnerable populations. There is also a pressing need to identify and validate predictive biomarkers of dietary responsiveness, and to establish clinical guidelines for the safe integration of nutrition-based therapies into standard oncologic care. For instance, a three-dimensional cube modeling approach was utilized to categorize patterns of macronutrient intake, resulting in the identification of 24 distinct dietary clusters. Of these, four specific clusters were significantly associated with reduced all-cause mortality [168]. In parallel, Hill number indices have been applied to quantify dietary biodiversity across different populations and contexts. Hill0—more commonly referred to as dietary species richness (DSR)—emerged as the index most strongly correlated with lower mortality rates. As such, DSR offers a promising metric for tracking progress toward more biodiverse and nutritionally balanced diets, while also serving as a sentinel marker for the adverse health effects linked to dietary monotony [155]. Without the incorporation of such quantitative tools and ecological frameworks, the rapid momentum driving the field of precision nutrigeroscience may risk outpacing its clinical translatability.

Given the broad and individualized impact of nutrition on health, it is imperative that dietary interventions be personalized according to the unique physiological and pathological context of each patient. For instance, high-fat diets such as KD may be contraindicated in patients with cardiovascular comorbidities [169]; carbohydrate-rich diets may be ill-suited for individuals with diabetes [170]; and CR or prolonged fasting may be inappropriate for underweight or cachectic patients [171]. Moreover, the nutritional demands and vulnerabilities of tumors vary widely depending on tissue of origin, metabolic phenotype, and immune environment. Glycolysis-dependent, “glucose-addicted” tumors may be more susceptible to carbohydrate restriction [172], whereas lipid-dependent cancers such as triple-negative breast and certain ovarian cancers may be more responsive to lipid-limiting strategies [173]. Similarly, tumors deficient in specific amino acid biosynthesis pathways may be rendered vulnerable through targeted amino acid restriction [174]. Importantly, the interplay between dietary regimens and therapeutic modalities must also be considered. Tumors that respond poorly to immunotherapy may derive enhanced benefit from diets that augment immunosurveillance, such as fasting or fasting-mimicking protocols [160]. Identifying such diet–therapy synergies represent a key frontier in the evolution of personalized cancer care.

Although dietary interventions are generally regarded as safe, emerging data suggest that caution is warranted, particularly in oncology. While these interventions hold substantial promise as non-genetic strategies to modulate cancer risk and treatment outcomes, their effects are not uniformly beneficial and remain incompletely understood. For example, CR—despite its tumor-suppressive effects in preclinical models—may induce adverse consequences that have yet to be fully characterized in humans [171]. Fasting has been shown to potentially exacerbate chemotherapy-induced cardiotoxicity, especially in frail or elderly individuals [175]. Protein restriction, although effective in attenuating tumor growth in experimental systems, may accelerate cancer-associated cachexia if not appropriately managed[176]. Probiotics, while widely promoted for their immunomodulatory properties, have been associated with episodes of bacteremia in immunocompromised patients [177]. Moreover, micronutrient supplementation has yielded inconsistent and sometimes harmful outcomes [[178], [179], [180]], with certain vitamins—such as B6 and E—linked to increased cancer recurrence in specific cohorts [28,178,179,181]. For example, vitamin E supplementation has been associated with increased recurrence risk in patients with non-muscle invasive bladder cancer [67], suggesting potential harm in certain settings. Conversely, another study reported that daily supplementation with 400 IU of vitamin E significantly reduced tumor recurrence in superficial bladder cancer, particularly among smokers [182]. In contrast, no significant association was observed between vitamin E intake and recurrence risk in breast cancer patients [183]. These contradictory findings highlight the context-dependent effects of vitamin E supplementation, influenced by cancer type, smoking status, nutritional background, and treatment regimen. Taken together, these findings underscore the need for rigorous, biomarker-driven, and ethically sound clinical frameworks that can guide the safe and effective deployment of dietary strategies in oncology.

## Conclusion and perspective

In this review, we summarize current knowledge on precision dietotherapy for cancer, with a focus on identifying specific components in diet that can impede progression of malignant disease and improve clinical manifestation. To address this, we propose a novel view of precision-nutrition medicine, named “Precision nutrigeroscience”.

There is incremental interest in an approach to nutritional therapy to selectively removes defined nutrients or supplement anticancer nutrition-relevant molecules for cancer treatment. To our knowledge, nicotinamide and vitamin D are potential substances functioning as chemoprevention in randomized clinical trials. Importantly, vitamins often mediate anticancer effects at high doses, well above those required for hypovitaminosis palliation. In parallel, the general public is often inundated with reports highlighting the potential benefits of plant-enriched elements, specifically polyphenols found in vegetables and fruits [184,185]. These healthy diets may contain low-abundance molecules favorably affecting cancerous ecosystem through initiating anti-tumor immunosurveillance and diminishing tumor-promoting inflammation. Several modern techniques like mass spectrometry and chromatographic fractionation are necessitated to identify hypoabundant molecules in healthy foods and screen the potential anti-carcinogenic compounds. It is worth noting that the therapeutic molecular species found in healthy diet perform as probiotics, which motivate the expansion of immunostimulatory bacteria in the intestines, and fulfil indirectly sanatory functions. Hence, there is promise in using adjunctive therapies that mimic and induce the physiological and molecular benefits of dietary interventions.

Although dietetical intervention is considered an effective treatment in pre-clinical model, there are several challenges regarding nutritive functions and their clinical practice. Firstly, future studies should concentrate on elucidating the mechanisms by which precise nutrimental components like carbohydrates, lipids, amino acids and microelements coordinate neoplastic development. Moreover, it is essential to explore the duration and circadian rhythm of dietary interventions, including dining times, curative cycles or endurance. It’s pivotal to elaborate the organically and molecularly synergistic effects by integration of dietetical therapy with other therapeutic interventions, such as exercise and sleep. Given to genetic variants in nutrition- sensitive pathways, it is necessary to define diverse genotypes for nutritive functions and the genetic disparity in individualized dietary intervention. The metabolic plasticity and flexibility within cancerous ecosystem suggest that dietary modulation will mediate cancer progression in distinct approaches. Therefore, precision dietotherapies are supposed to consider the metabolic diversity and mutability in tumor ecological environment. Due to the heterology in individuals' responses to diet-based therapeutic strategies, there is a requirement to develop biomarker panels and optimize nutritive interventions. Demographic factors like sex and age necessitate to be taken into consideration in diet-intervening trials. Successive diet-based therapeutics require incorporation with multi-omics profiles and systems biology to elucidate their pivotal roles in maximizing therapeutic effects.

We hope that in the future, more consideration will be taken into the dietary recommendations for cancer patients, and it becomes a priority to explore how modifying the diet is able to potentially delay disease progression and improve the response to cancer therapies. A paradigm shift in cancer treatment will be gestated via prioritizing research in this direction.

## Compliance with ethics requirements

This article does not involve any studies with human or animal subjects.

## Declaration of competing interest

The authors declare that they have no known competing financial interests or personal relationships that could have appeared to influence the work reported in this paper.
